# Enhancing Psychological and Physical Fitness Factors of Korea Middle School Students by Introducing Rope Skipping

**Published:** 2018-12

**Authors:** Tae-Jun CHON, Dong Jun SUNG, Jae-Youn JEON, Jung-Taek SHIN

**Affiliations:** 1. School of Sports, Soongsil University, Seoul, Korea; 2. Division of Sport and Health Science, College of Biomedical and Health Science, Konkuk University, Chungju, Korea; 3. Korea Institute of Sport Science, Seoul, Korea; 4. Dept. of Physical Education, Dongeui University, Busan 47340, Korea

## Dear Editor-in-Chief

In Korea, it is highly competitive to enter next-level schools. Korean adolescents who are in this competition have low level of physical activity with high frequency of psychological instability (1). Both physical health and mental state are important for adolescents. There is a strong correlation between these two factors (1). In addition, physical activity is known to have positive effect on adolescents’ self-esteem, happiness, and academic achievement (2).

Nonetheless, the rate of adolescents’ participation in moderate to high intensity physical activity is low (12.6 %) in Korea (3). Such low participation rate of physical activity could negatively influence their emotional, mental, and physical health. On the other hand, regular exercise participation of adolescents can effectively improve their academic achievement with various health benefits. Therefore, an effective exercise program should be introduced in Korea to encourage regular exercise participation of adolescents.

Among various forms of exercise, rope skipping is an activity that moves both upper and lower limbs actively. During rope skipping, leg jumping and arm rotation occur repeatedly. Balance and coordination are both needed for successful rope jumping. In addition, rope skipping has positive effects on the cardiorespiratory system (4). Rope skipping intervention can increase the physical activity time of adolescents, making their participation in class easier (5). The aim of this study was to determine whether rope skipping could affect middle school students psychologically and physiologically.

Overall, 71 male middle school students in Seoul, South Korea (age: 13.80 ± 0.40 yr, height: 163.44± 8.43 cm, weight: 56.69 ± 12.01 kg) without musculoskeletal injuries or other diseases participated. Psychological inventories were used to evaluate psychological factors such as stress, self-esteem, happiness, leisure efficiency, and leisure satisfaction (Cronbach’ *a*=0.732–0.877). Physiological and physical fitness factors including salivary cortisol (μg/dL, a stress hormone), shuttle run (repetition/rep, a cardiopulmonary fitness factor), and 50 m of sprint (second, an agility factor). Rope skipping intervention consisted of a personal program and a group program. It was performed three sessions per week (30 min per session). Exercise intensity was 13 (somewhat hard) of Borg’s rating of perceived exertion scale. The exercise program was conducted under the supervision of class teacher.

All statistical analysis was performed using paired t-test of SPSS ver. 19.0 for windows (Chicago, IL, USA). If any significant differences were observed, effect size (ES, *d*) (6) was calculated, with *d*=0.2, *d*=0.4, and *d*=0.8 indicating small, medium, and large effects, respectively. Statistical significance was set at *P*<0.05.

Stress scores before and after rope jumping intervention were 2.75±1.26 and 2.37 ± 1.05, respectively, showing significant difference (*t*=3.609, *P*=0.001, *d*=0.32). Self-esteem scores before and after intervention were 3.50±0.91 and 3.82±0.80, respectively, showing significant difference (*t*= −3.739, *P*<0.001, *d*=0.26). Happiness scores before and after intervention were 6.73±1.46 and 7.22±1.47, respectively, with significant difference (*t*= −2.679, *P*=0.009, *d*=0.23). Leisure efficiency scores before and after intervention were 3.52±0.92 and 3.77±0.89, respectively, with significant difference (*t*= −2.249, *P*=0.028, *d*=0.19). Leisure satisfaction scores before and after intervention were 3.60±0.89 and 3.73±0.91, respectively, without significant difference (*P*>0.05).

Salivary cortisol level before rope skipping was 0.21±0.13 μg/dL. It was significantly decreased to 0.13±0.05 μg/dL (*t*=2.294, *P*=0.045, *d*=0.58) after rope skipping intervention. Cardiopulmonary physical fitness based on shuttle run was significantly enhanced (31.73±12.90 rep before intervention vs. 34.91±12.59 rep. after intervention, *t*= −6.826, *P*<0.001, *d*=0.17). Results of the 50 m sprint test before and after intervention were 8.93±1.02 sec and 8.87±1.00 sec (*t* = 3.128, *P*=0.003, *d*=0.04), respectively. Our results suggest that school-based rope skipping has positive influence on psychological and physiological factors of adolescents. To have more effective exercise programs in schools, rope skipping should be included.
